# Sustainable carbon sources for microbial organic acid production with filamentous fungi

**DOI:** 10.1186/s13068-017-0930-x

**Published:** 2017-10-23

**Authors:** Stefan Dörsam, Jana Fesseler, Olga Gorte, Thomas Hahn, Susanne Zibek, Christoph Syldatk, Katrin Ochsenreither

**Affiliations:** 1 Technical Biology, Institute of Process Engineering in Life Sciences, Karlsruhe Institute of Technology (KIT), Engler-Bunte-Ring 3, Karlsruhe, 76131 Germany; 2 Industrial Biotechnology, Department of Molecular Biotechnology, Fraunhofer Institute for Interfacial Engineering and Biotechnology (IGB), Stuttgart, Germany

**Keywords:** *Aspergillus oryzae*, *Rhizopus delemar*, Malic acid, Malate, Fermentation, Organic acid, Lignocellulose, Organosolv, Levoglucosan, Filamentous fungi

## Abstract

**Background:**

The organic acid producer *Aspergillus oryzae* and *Rhizopus delemar* are able to convert several alternative carbon sources to malic and fumaric acid. Thus, carbohydrate hydrolysates from lignocellulose separation are likely suitable as substrate for organic acid production with these fungi.

**Results:**

Before lignocellulose hydrolysate fractions were tested as substrates, experiments with several mono- and disaccharides, possibly present in pretreated biomass, were conducted for their suitability for malic acid production with *A. oryzae.* This includes levoglucosan, glucose, galactose, mannose, arabinose, xylose, ribose, and cellobiose as well as cheap and easy available sugars, e.g., fructose and maltose. *A. oryzae* is able to convert every sugar investigated to malate, albeit with different yields. Based on the promising results from the pure sugar conversion experiments, fractions of the organosolv process from beechwood (*Fagus sylvatica*) and *Miscanthus giganteus* were further analyzed as carbon source for cultivation and fermentation with *A. oryzae* for malic acid and *R. delemar* for fumaric acid production. The highest malic acid concentration of 37.9 ± 2.6 g/L could be reached using beechwood cellulose fraction as carbon source in bioreactor fermentation with *A. oryzae* and 16.2 ± 0.2 g/L fumaric acid with *R. delemar*.

**Conclusions:**

We showed in this study that the range of convertible sugars for *A. oryzae* is even higher than known before. We approved the suitability of fiber/cellulose hydrolysate obtained from the organosolv process as carbon source for *A. oryzae* in shake flasks as well as in a small-scale bioreactor. The more challenging hemicellulose fraction of *F. sylvatica* was also positively evaluated for malic acid production with *A. oryzae*.

## Background

The majority of industrial processes for the production of chemicals, materials, and also energy are still based on fossil fuels, especially coal, and crude oil. To gain independence from these raw materials, sustainable sources, and environmental friendly methods to produce relevant platform chemicals are becoming increasingly important. Suitable candidates meeting these requirements are different dicarboxylic acids, due to the application in the synthesis of various polymers which was summarized by Lee et al. [1]. In particular, the C4 dicarboxylic acids malic acid, fumaric acid, and succinic acid were selected in 2004 by the US Department of Energy to be among the 12 most important platform chemicals available from biomass. Based on these three platform chemicals, numerous other chemicals and fine chemicals as well as polymers may be synthesized that in turn can be used in food or the pharmaceutical industry [2]. Currently, malic and fumaric acid are predominantly synthesized from petroleum [3, 4]. For the biotechnological production of these organic acids, some species of the genus *Aspergillus* and *Rhizopus* appear to be promising. These species can produce considerable amounts of malic acid and fumaric acid via the reductive TCA cycle under certain stress conditions [5–7]. Production processes have been further optimized in the last years, so that in fermentations with 120 g/L glucose as carbon source malic acid concentrations of 113 g/L were achieved with *Aspergillus flavus* [8]. Because of the production of aflatoxins, this fungus is not suitable for industrial production of malic acid. The production of malate by the close relative *Aspergillus oryzae*, which is not producing aflatoxins, has also been investigated. Through metabolic engineering of *A. oryzae* strain NRRL 3488, malic acid concentrations of 154 g/L were produced from 160 g/L glucose [9]. So far, a biotechnological production of malic acid is not industrially established due to the high process costs compared to the conventional chemical synthesis. However, keeping in mind the industrial production process of citric acid by *Aspergillus niger* with an annual production of 1.6 million tons in 2012 [10] a biotechnological process for malic acid seems to be feasible. In order to establish an industrial production of malic acid using various microorganisms from an economic perspective, the processes need to be further optimized to obtain higher yields and improved cost efficiency. As part of the “food or fuel”-debate, a biotechnological malic acid production based on alternative carbon sources not competing with food or feed production would be desirable. Lignocellulose, as an abundant renewable resource of the second generation, is easily available but its microbial accessibility is a challenge. It could be shown that *A. oryzae* is also able to convert alternative carbon sources to malic acid like glycerol and xylose [11] which is also a part of lignocellulosic material. Thus, alternative carbon sources based on lignocellulose, such as hydrolysates from lignocellulose separation or pyrolysis oils from thermal treatment of lignocellulosic biomass, are possibly suitable for malate production by *Aspergilli*. Several components of pyrolysis oil, like acetic acid [12] and the pyrolytic sugar levoglucosan, are promising substrates for *A. oryzae*. Untreated pyrolysis oil is not a suitable carbon source [13–16]. Compared to this, the organosolv process is an attractive method for separation of wooden biomass into the three main components of lignocellulose: Cellulose, hemicellulose and lignin. The enzymatic saccharification of cellulose leads to a glucose-rich fraction, whereas a xylose-rich fraction results from hemicellulose [17]. The challenge of using lignocellulose hydrolysates is on one hand the potential formation of toxic compounds during the fractionation process, mainly phenols from lignin, furfural, and hydroxymethylfurfural (HMF) from cellulose and hemicellulose [18]. On the other hand, the pretreatment process results in the formation of xylose oligomers in the supernatant which cannot be adequately enzymatic hydrolyzed afterwards due to unfavorable conditions and degradation products present. Both aspects make especially the xylose-containing fraction to the more challenging substrate. The aim of this study is the evaluation of pyrolytic sugar, different pretreated and post-treated fractions from the organosolv process from beechwood (*Fagus sylvatica)*, and *Miscanthus giganteus* as substrate for the fermentative malic acid production with the filamentous fungus *A. oryzae*.

## Methods

### Chemicals

All chemicals were either purchased from Sigma-Aldrich (Munich, Germany) or Roth (Karlsruhe, Germany).

### Hydrolysate preparation

Different fractions were obtained by the organosolv process incubating the chopped raw material at high temperatures (> 140 °C) in aqueous ethanol solution with small amount of H_2_SO_4_ as catalyst. The fiber fraction, mainly containing the cellulose and a part of hemicellulose, was directly subjected to enzymatic hydrolysis after washing. The supernatant of the organosolv process was further processed to isolate the lignin and to utilize the carbohydrates from hemicellulose for fermentative purposes. Carbohydrate, acid, and toxic compound content of the resulting solution was quantified via HPLC (see Sluiter et al. [19] for further description). Processing of the residual fractions was carried out as follows.

#### Beechwood

##### Fiber (cellulose) fraction

Enzymatic hydrolysis of the fiber was performed at a temperature of 50 °C with a 10% (w/v) suspension for 24 h. For stirrer description see Ludwig et al. [20]. pH of the suspension was adjusted to pH 4.8 during hydrolysis using a concentrated NaOH solution. Enzyme addition (0.06 g Cellic^®^ CTec3 and 0.0025 g Cellic^®^ HTec3 per g cellulose) started the reaction. The solid material was afterwards removed applying an extruder press. The successive evaporation of the filtrate resulted in the mono- and disaccharide concentrations shown in Table 1. 108.7 g of this fraction was used for fermentation purposes.

**Table 1:** Composition of the different Lignocellulose fractions from beechwood and *Miscanthus*

	Beechwood hemicellulose fraction (g/L)	Beechwood fiber (cellulose) fraction (g/L)	*Miscanthus* fiber (cellulose) fraction (g/L)
Ethanol	1	0	0
Acetic acid	15	0	0.2
Cellobiose	0	67	0
Glucose	20	609	102
Xylose monomer	100	179	25
Xylose oligomer	310	0	0
Rhamnose	27	0	0
Arabinose	18	0	0.2

##### Hemicellulose fraction

After removal of the biomass, evaporation of the residual ethanol was performed to precipitate the lignin and to concentrate the carbohydrates. Enzymatic hydrolysis was not performed with this fraction. The compounds shown in Table 1 could be identified by total hydrolysis and subsequent chromatographic analysis. 99.5 g of this fraction was used for fermentation.

#### *Miscanthus* fiber

Enzymatic hydrolysis of the fiber was performed at a temperature of 50 °C with a 10% w/v suspension for 24 h. For stirrer description see Ludwig et al. [20]. pH of the suspension was adjusted to pH 4.8 using a concentrated NaOH solution during hydrolysis. Enzyme addition (0.06 g Cellic^®^ CTec2 per g cellulose, 0.006 g Cellic^®^ HTec2 per g cellulose) started the reaction. Residual solid material was removed after hydrolysis by centrifugation for 15 min at 4696*g*. Succeeding concentration of the supernatant via evaporation resulted in the concentrations shown in Table 1. For shake flask cultivation, the solution was diluted to 100 g/L carbon sources and the salts for main-culture medium were added.

### Fungi and media

The fungal strains *A. oryzae* DSM 1863 and *R. delemar* DSM 905 were obtained from DSMZ strain collection (Deutsche Sammlung von Mikroorganismen und Zellkulturen GmbH, Braunschweig, Germany) and treated like described in Dörsam et al. [13]. *A. oryzae* was grown on minimal medium (MM) for *Aspergillus* spec. [21]: 6 g/L NaNO_3_, 0.52 g/L KCl, 0.52 g/L MgSO_4_·7H_2_O, and 1.52 g/L KH_2_PO_4_. The pH was set to 6.5 with NaOH. 10 g/L glucose, 2 mL of 1000× Hutner’s Trace Elements, and 15 g/L agar were added afterwards. 1000× Hutner’s Trace Element solution consists of 5 g/L FeSO_4_·7H_2_O, 50 g/L EDTA-Na_2_, 22 g/L ZnSO_4_·7H_2_O, 11 g/L H_3_BO_3_, 5 g/L MnCl_2_·4H_2_O, 1.6 g/L CoCl_2_·6H_2_O, 1.6 g/L CuSO_4_·5H_2_O, and 1.1 g/L (NH_4_)_6_Mo_7_O_24_·4H_2_O, pH 6.5 [21]. *R. delemar* was grown on modified supplemented agar (SUP): 10 g/L glucose, 0.5 g/L yeast extract, 4 g/L KH_2_PO_4_, 0.9 g/L K_2_HPO_4_, 4 g/L NH_4_Cl, 0.25 g/L MgSO_4_·7H_2_O. The pH was set to 6.5 with NaOH.

For conidia collection, *A. oryzae* was grown on high-salt minimal medium [22] which additionally contains 22.37 g/L KCl. For spore collection, *R. delemar* was grown on malt extract agar (MEA): 30 g/L malt extract, 3 g/L peptone, 15 g/L agar. The conidia and spores were harvested with 50% glycerol from plates that were incubated for 5 days at 30 °C and filtrated with Miracloth (Calbiochem). The spore/conidia solution was diluted to a concentration of 1 × 10^7^ (spore/conidia)/mL and stored at − 80 °C.

Malic acid production was accomplished in a two-step process with a pre-culture and a main-culture. The pre-culture medium consists of 40 g/L glucose monohydrate, 4 g/L (NH_4_)_2_SO_4_, 0.75 g/L KH_2_PO_4_, 0.98 g/L K_2_HPO_4_·3H_2_O, 0.1 g/L MgSO_4_·7H_2_O, 0.1 g/L CaCl_2_·2H_2_O, 5 mg/L NaCl, and 5 mg/L FeSO_4_·7H_2_O. Main-culture medium contains the corresponding carbon source, in equivalent carbon amounts as in control medium. The control contains 120 g/L glucose monohydrate, 1.2 g/L (NH_4_)_2_SO_4_, 0.1 g/L KH_2_PO_4_, 0.17 g/L K_2_HPO_4_·3H_2_O, 0.1 g/LMgSO_4_·7H_2_O, 0.1 g/L CaCl_2_·2H_2_O, 5 mg/L NaCl, and 60 mg/L FeSO_4_·7H_2_O. To keep the pH level above 5.5 during fermentation, 90 g/L CaCO_3_ powder was added to the main-culture medium. All media were sterilized by autoclaving.

### Organic acid production

For *A. oryzae* pre-culture, 100 mL of pre-culture medium in a 500-mL baffled Erlenmeyer shake flasks was inoculated with 2 × 10^7^ conidia. The flasks were incubated at 100 rpm and 30 °C for 24 h in a rotary shaker. After incubation, pre-culture medium was removed by washing the fungal pellets twice and resuspending in 100 mL water. 100 mL of the main culture was transferred to 500-mL Erlenmeyer shake flasks and 9 g/L sterile CaCO_3_ powder added. The flasks were inoculated with 10% (*v*/*v*) of washed pre-culture and incubated at 120 rpm and 32 °C for 7 days.

For *R. delemar* pre-culture, 100 mL of pre-culture medium was filled into 500-mL baffled Erlenmeyer shake flasks and inoculated with 1 × 10^7^ spores. The flasks were incubated at 100 rpm and 35 °C for 30 h in a rotary shaker. To remove the pre-culture medium, fungal pellets were washed twice and resuspended in 100 mL water. Every cultivation was done in triple approach.

For the bioreactor cultivations, 1.5 L of main-culture medium was used. Additionally, 120 g CaCO_3_ powder for pH regulation and 200 μL of antifoam reagent (Contraspum A 4050 HAC, Tschimmer und Schwarz) were added before autoclaving. The bioreactor was inoculated with the fungal biomass of two pre-culture flasks (suspended in 100 mL water) for *A. oryzae* and with the biomass of five pre-culture flasks (suspended in 100 mL water) for *R. delemar*. Every fermentation was done in double approach. The fermentation was carried out in a small-scale bioreactor (vessel volume 2.0 L) Minifors (Infors, Switzerland) at 35 °C, an aeration rate of 0.5 vvm, and a stirrer speed of 300 rpm. A Rushton turbine with a diameter of 46 mm was chosen as stirrer. Every cultivation was done in double approach.

### Organic acid and carbohydrate analytics

For the malic acid quantification with HPLC, fermentation broth samples were pretreated and analyzed as described in Ochsenreither et al. [11] with slight modifications. Malic acid was released from precipitated calcium malate by mixing 1 mL sample with 1 mL of 3 M H_2_SO_4_ and 3 mL of water incubating the homogenate at 80 °C for 20 min. 1 mL of the mixture was transferred to a 1.5-mL Eppendorf tube and centrifuged in a table top centrifuge for 5 min at 20,000×*g*. The supernatant was used for HPLC analysis. The analysis was performed with a standard HPLC device (Agilent 1100 Series, Agilent, Germany) equipped with a 15-cm reversed phase column (Synergi™4 μm Fusion-RP 80 Å, LC Column 150 × 4.6 mm, Phenomenex, Aschaffenburg, Germany) at 30 °C. Mobile phase solution A was methanol, and solution B was 20 mM KH_2_PO_4_, pH 2.5. The flow rate was 1 mL/min and a gradient was used for the separation of organic acids: 0–0.5 min 100% eluent B, 0.5–10 min linear increase of eluent A from 0 to 10%, 10–12 min decrease of eluent A back to 0%, and 12–14 min again 100% eluent B. The injection volume was 10 μL and the detection was performed with a UV detector at a wavelength of 220 nm. Standards were purchased from Sigma-Aldrich (Munich, Germany) and used for peak identification and calibration. Linear detection ranged from 0.1 to 5 g/L malic acid and 0.02–0.5 g/L fumaric acid.

For the carbohydrate quantification with HPLC, fermentation broth samples were pretreated and analyzed as described by Buchholz et al. [23] with slight modifications described by Siebenhaller et al. [24]. A protocol for phosphate precipitation was applied before measurement. 45 µL 4 M NH_3_ and 100 µL 1.2 M MgSO_4_ were added to 1000 µL sample and subsequently centrifuged for 5 min at 20,000×*g* after 5 min of incubation. 500 µL supernatant was then mixed with 500 µL 0.1 M H_2_SO_4_ and incubated for 15 min. After the final centrifugation step of 15 min at 20,000×*g*, the supernatant was used for HPLC analysis. The analysis was performed with a standard HPLC device (Agilent 1100 Series, Agilent, Germany) with a Rezex ROA organic acid H+ (8%) column (300 by 7.8 mm, 8 m; Phenomenex) and a Rezex ROA organic acid H+ (8%) guard column (50 by 7.8 mm). Separation was performed under isocratic conditions at 50 °C (column temperature) for 45 min with 5 mM H_2_SO_4_ as the mobile phase at a constant flow rate of 0.5 mL/min. Detection of carbohydrates was achieved via a refractive index detector (Agilent 1200 series, Agilent, Germany).

### Data analysis

Carbon source consumption and malic acid production was fitted using a logistic equation with four parameters with a scientific data analysis and graphing software (Sigma Plot 9.0, Systat, San Jose, USA). The used equation was

$$y\left( x \right) \, = \, y_{0} + \frac{a}{{ 1 { + }\left( {\frac{x}{{x_{0} }}} \right)^{b} }}.$$

The four parameters are the following: *y*_0_ indicates the minimum concentration of the carbon source/product; a indicates the maximum carbon source/product concentration; *x*_0_ indicates the process time when half of the carbon source amount is consumed or half of the maximum product concentration is produced; *b* is a shape parameter and difficult to explain biologically [25].

Consumption and production rates were calculated as the derivation of this equation.

## Results

### Pure sugar conversion experiments

The main challenge of using non-food sugars in biotechnological applications is firstly the ability of the respective organism to metabolize different sugars in general, and secondly, especially for pretreated lignocellulosic material, the tolerance concerning degradation products formed during the pretreatment process.

Therefore, several mono- and disaccharides possibly contained in alternative carbon sources were tested for their suitability as substrates for malic acid production with *A. oryzae*. This includes the anhydrosugar levoglucosan, formed during flash pyrolysis, carbohydrates contained in lignocellulose like glucose, galactose, mannose, arabinose, xylose, ribose, and cellobiose, as well as cheap and easy available sugars like fructose and maltose. The results are summarized in Table 2.

**Table 2:** Calculated parameters of tested carbon sources in shake flask cultivation of *A. oryzae*. Flasks were incubated at 32 °C for 168 h

	Carbon source	g/L (carbon source)^a^	ϲ (malate) g/L^b^	*Y*_P/S_ g/g^b^	*Q*_*p*_ max g/(L * h)^c^	Time span of *Q*_*p*_ max h^c^	*Q*_s_ max g/(L * h)^c^	Time of *Q*_*s*_ max h^c^	*Q*_*p*_ overall g/(L * h)^b^
(Anhydro-) hexoses	Glucose	109	40.5 ± 3.7	0.65	0.41	64.13 – 79.31	0.61	0 – 1.13	0.24
	Fructose	109	24.8 ± 1.9	0.63	0.22	107.63 – 131.25	0.39	61.69 – 73.5	0.15
	Galactose	109	1.8 ± 0.5	0.06	0.015	164.72 – 168	0.25	0 – 1.97	0.01
	Mannose	109	32.8 ± 0.5	0.69	0.29	108.94 – 134.53	0.40	6.56 – 34.78	0.19
	Levoglucosan	98.2	17.2 ± 1.7	0.34	0.14	103.03 – 143.72	0.36	0 – 10.5	0.1
Pentoses	Arabinose	109	7.2 ± 2.9	0.22	0.06	108.94 – 140.44	0.42	0 – 1.97	0.04
	Ribose	109	20.7 ± 5.7	0.45	0.18	110.25 – 148.31	0.33	0 – 10.5	0.12
	Xylose	109	24.3 ± 3.3	0.49	0.20	97.78 – 123.38	0.31	139.78 – 168	0.14
Disaccharides	Maltose	103.6	34.1 ± 10.8	0.34^d^	0.30	96.47 – 110.25	1.57^d^	56.44 – 60.38^d^	0.2
	Cellobiose	103.6	8.8 ± 1	0.14^d^	0.10	101.06 – 130.59	0.65^d^	0 – 0.66^d^	0.05
Mixed	75% Glucose, 25% Xylose	81.8 27.3	29.4 ± 1.9	0.38	0.29	47.81 – 67.50	1.02	0 – 0.56	0.2
	25% Glucose, 75% Xylose	27.3 81.8	31.9 ± 0.3	0.59	0.31	140.63 – 149.63	0.90	0 – 0.56	0.22

ϲ (Malate) = final product concentration; *Y*_P/S_ = substrate specific yield; *Q*_*p*_ max = maximal volumetric production rate; *Q*_s_ max = maximal volumetric consumption rate

^a^Weighed amount

^b^Measured values

^c^Calculated values

^d^Based on disaccharide cleavage

*Aspergillus oryzae* is able to convert every tested sugar to malate, albeit with different yields. The hexose fructose and the disaccharide maltose, not derived from lignocellulosic material, turned out to be a very promising substrate.

The highest malic acid titer was achieved with glucose (40.5 ± 3.7 g/L). This approach is also used as the control cultivation. Subsequently, maltose led to the second highest malic acid concentration of 34.1 ± 10.8 g/L. Cultivations with mannose and the two testes mixture of glucose and xylose resulted in final product concentrations around 30 g/L. Around 20 g/L could be achieved by using fructose (24.8 ± 1.9 g/L), levoglucosan (17.2 ± 1.7 g/L), ribose (20.7 ± 5.7 g/L), and xylose (24.3 ± 3.3 g/L) as sole carbon source (Table 2). Only three of the tested carbon sources led to a final malic acid concentration below 10 g/L, namely cellobiose, arabinose, and galactose. Product yields correlate slightly with malic acid titers. The theoretical yields of (anhydro)hexoses are 2 mol organic acid per mol carbon source which is 1.49 g/g for malic acid and 1.29 g/g fumaric acid with hexoses and 1.65 g/g malic acid with levoglucosan. For disaccharides, 4 mol organic acid per mol carbon source (1.57 g/g) and for pentoses 1.67 mol organic acid per mol carbon source (1.49 g/g) were observed [11].

The highest yield was achieved with mannose with 0.69 g/g which corresponds to 46% of the maximum theoretical yield. The control approach with glucose resulted in a yield of 0.65 g/g (44%), the mixture of 25% glucose and 75% xylose in a yield of 0.59 g/g (40%) and with fructose in a yield of 0.63 g/g (42%). The yields for all of the other tested carbon sources were below 0.5 g/g. Cultivation with xylose (0.49 g/g; 33%), ribose (0.45 g/g, 30%), the mixture of 75% glucose and 25% xylose (0.38 g/g, 26%), levoglucosan (0.34 g/g; 21%), maltose (0.34 g/g; 22%), and arabinose (0.22 g/g; 18%) resulted in concentrations in the middle range. Lowest yields were achieved for cellobiose with 0.14 g/g (9%) and galactose with 0.06 (4%) g/g.

**Fig. 1:**
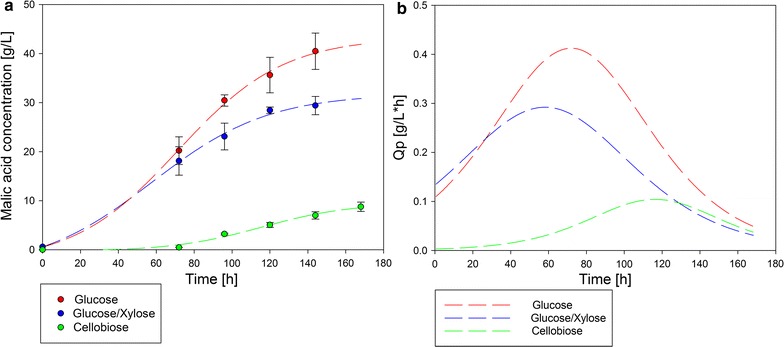
Examples of malic acid formation (**a**) and volumetric production rates (**b**) during cultivation of *A. oryzae* DSM 1863 by using different carbon sources. Flasks were incubated at 32 °C for 168 h. *Q*_*p*_ = volumetric production rate

Production rates were calculated as derivation of malic acid concentration fit (sigmoidal, four parameters) during cultivation time. The malic acid concentration as well as the corresponding volumetric production rates during cultivation with glucose, mixture of 75% glucose and 25% xylose and cellobiose are exemplary shown in Fig. 1.

The volumetric production rates of malic acid differ widely between the different carbon sources. The production rate plotted against the cultivation time results in a parabolic curve. Their peak corresponds to the time point of the maximum production rate.

Highest maximal rate could be observed during cultivation with glucose (0.41 g/(L * h)) between 64.13 and 79.31 h of cultivation. Production rates of both glucose and xylose mixtures, mannose, and maltose were approximately 0.3 g/(L * h). Using fructose as carbon source resulted in a maximal production rate of 0.22 g/(L * h) and was observed during the cultivation period from 107.63 to 131.25 h. For xylose, ribose, levoglucosan, and cellobiose, the highest volumetric production rates were 0.20, 0.18, 0.14, and 0.10 g/(L * h). The lowest maximal production rates could be observed with arabinose (0.06 g/(L * h)) between cultivation hour 108.94 and 140.44 and with galactose (0.015 g/(L * h)).

Maximal volumetric consumption rates of carbon sources did not correlate with maximal production rates of malic acid. The highest consumption rate was detected for maltose (1.57 g/(L * h)). The second highest consumption rate could be observed with the mixture of 75% glucose and 25% xylose of 1.02 g/(L * h) followed by 25% glucose and 75% xylose of 0.90 g/(L * h). Cellobiose and the control approach glucose showed the maximum of consumption rate in an early stage of cultivation (0.65 g/(L * h) and 0.61 g/(L * h)). By using arabinose, mannose, and fructose, the maximal consumption rate of about 0.40 g/(L * h) was achieved. Ribose, xylose, and finally galactose showed the lowest maximal consumption rates of about 0.30 g/(L * h).

The pure sugar conversion experiment showed the suitability of several sugars. By focusing on organosolv-pretreated lignocellulose fractions, the most important sugars are cellobiose, glucose, and xylose, whereas galactose, ribose, arabinose, and mannose only occur in trace amounts. Besides the sugars, toxic compounds formed during organosolv process derived from sugars and lignin can be a major problem for many organisms. The limiting inhibiting concentrations for several typical impurities derived from lignin as well as some impurities derived from sugars are described in earlier studies by our group [13] but do not include hydroxymethylfurfural (HMF), the most common impurity. To investigate the tolerance during malic acid production phase, various concentrations of HMF were added to main-culture medium and malic acid concentration was measured during cultivation period. The analyzed HMF contents were 0, 0.1, 0.15, and 0.2%. No inhibiting influence could be observed for all concentrations tested (data not shown).

### Cultivation of *A. oryzae* with different lignocellulose-derived fractions

**Fig. 2:**
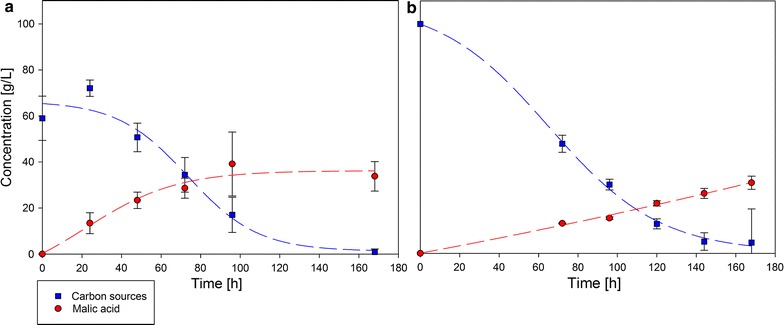
Carbon source and malic acid concentration during cultivation with *A. oryzae* DSM 1863 and cellulose/fiber hydrolysates from *F. sylvatica* (**a**) and *M. giganteus* (**b**). Flasks were incubated at 32 °C for 168 h

Because of the promising results from the pure sugar conversion experiments, fractions of the organosolv process were further assessed as carbon source for cultivation. For this study, fractions of two different plants were used (*F. sylvatica* and *M. giganteus*). For each plant, fibers were separated and pretreated as described in the materials section. Both cellulose fractions were saccharified and concentrated. For *F. sylvatica* cellulose hydrolysate, no HMF could be detected. 108.7 g of beechwood fraction was used for cultivation. *Miscanthus giganteus* hydrolysate solution was diluted to 100 g/L carbon source. During cultivation in shake flasks, carbon source concentration and product formation was determined. The curves are shown in Fig. 2.

During cultivation with *F. sylvatica* fiber hydrolysate, the detectable carbon sources (glucose and xylose) decreased from 59 to 0.9 g/L, whereas the malic acid concentration increased from 0 to 33.8 ± 6.4 g/L corresponds to an overall production rate of 0.2 g/(L * h). This results in a yield related to glucose and xylose of 0.58 g/g (39%). Shake flasks with *M. giganteus* cellulose hydrolysate showed a decrease of carbon source concentration (glucose and xylose) from 100 to 4.7 g/L and a final malic acid titer of 30.8 ± 2.9 g/L which results in a yield of 0.32 g/g (22%) and an overall production rate of 0.18 g/(L * h). The highest volumetric production rate (0.54 g/(L * h)) was determined after 18.38 h and decreased after 24.28 h for beechwood cellulose hydrolysate. For *M. giganteus* cellulose hydrolysate, the maximal production rate of 0.21 g/(L * h) was reached after 156.84 h until the end of cultivation. Compared to this, maximal volumetric consumption rate of 0.92 g/(L * h) were calculated from cultivation hour 71.53 until hour 75.47 for beechwood and for *M. giganteus* from 60.38 to 70.88 h (0.97 g/(L * h)).

**Fig. 3:**
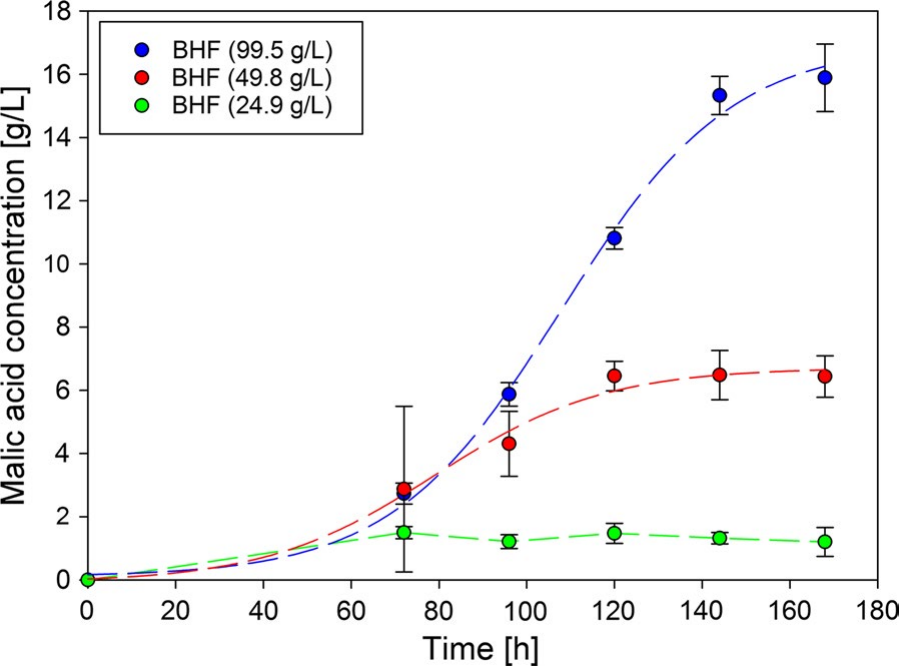
Malic acid concentration during cultivation of *A. oryzae* DSM 1863 with hemicellulose fraction from beechwood (BHF) in three different concentrations. Flasks were incubated at 32 °C for 168 h

In contrast to the fiber hydrolysate, the hemicellulose fraction of *F. sylvatica* was not saccharified. The fraction was used as the carbon source in the main culture in shake flask cultivations. The monosaccharide content is formed during the harsh organosolv process conditions. As the major impurity, 4.5 g/L furfural could be detected in this fraction. Further impurities derived from lignin and sugars were expected. HPLC measurements showed that they only occur in trace amounts. Besides furfural, acetic acid (15 g/L) must be seen as an impurity, but is also a possible carbon source for *A. oryzae* for malic acid production [12]. Because of the impact of impurities, three different amounts of *F. sylvatica* hemicellulose were used to observe possible inhibition effects. Malic acid concentrations during cultivation time were measured and curves are shown in Fig. 3. The amount of beechwood hemicellulose fraction (BHF) that was used correlated to the corresponding amount of carbon in the control approach with glucose: 99.5, 49.8, and 24.9 g.

With all concentrations, a lag phase of about 48 h was observed before malic acid production started which did not occur in the other cultivations with refined sugars. Using 99.5 g/L beechwood hemicellulose fraction, a malic acid titer of 15.9 ± 1.1 g/L could be achieved. A concentration of 49.8 g/L resulted in a final product concentration of 6.4 ± 0.7 and 24.9 g/L carbon source resulted in 1.2 ± 0.5 g/L malic acid after a cultivation time of 168 h. Maximal volumetric production rate (0.22 g/(L * h)) could be observed during 103.69 and 112.22 h for 99.5 g/L and between 72.19 and 85.97 h for 49.75 g/L (0.09 g/(L * h)). The amount of malic acid produced from a concentration of 24.9 g/L was low. The production rate between the first two samples can be calculated and is approximately 0.02 g/(L * h). Because of the complexity of the hemicellulose hydrolysate, carbohydrates could not be fully quantified during cultivation. Related to all known carbon sources (glucose, xylose, oligoxylose, rhamnose, arabinose, and acetic acid) at the beginning, the yields were 0.42, 0.34, and 0.13 g/g with decreasing amount of hemicellulose fraction. The overall production rates were 0.09, 0.04, and 0.007 g/(L * h).

### Scale-up fermentation of organosolv fractionated lignocellulose with *A. oryzae*

**Fig. 4:**
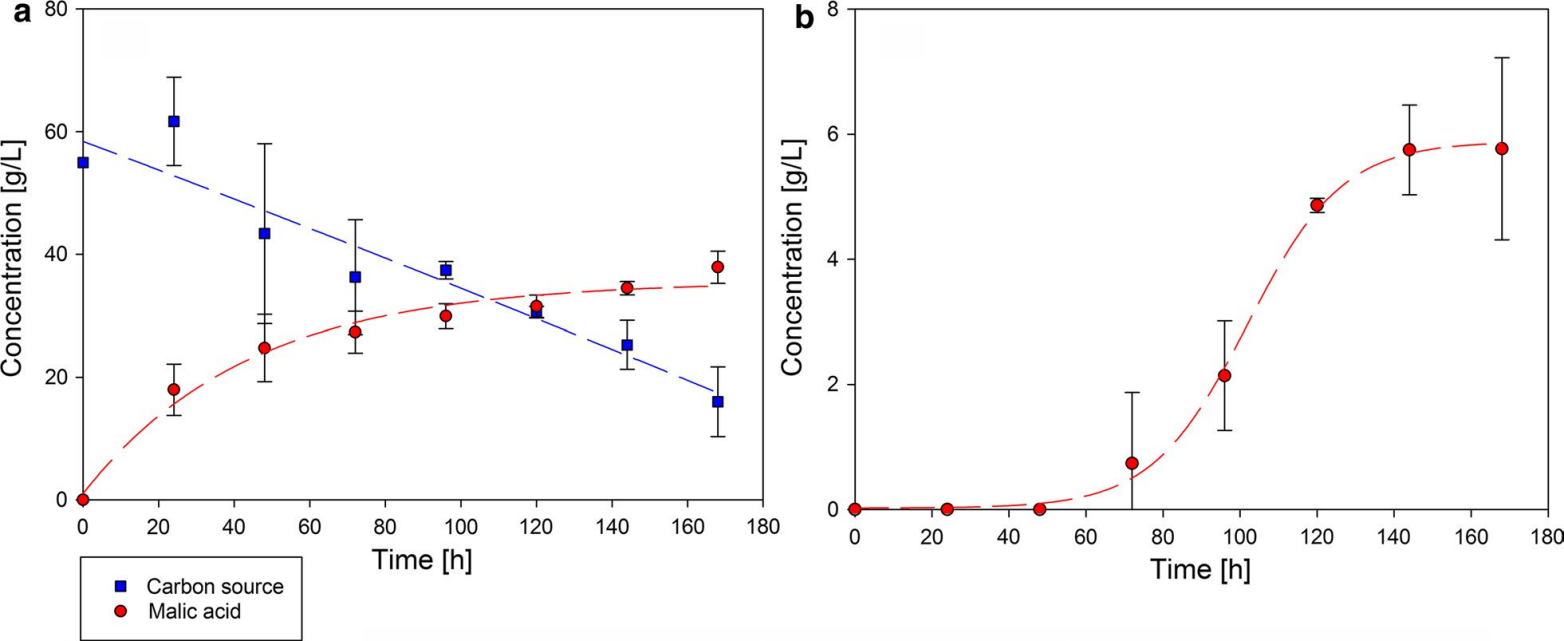
Carbon source and malic acid concentration by fermentation of *A. oryzae* DSM 1863 with cellulose/fiber hydrolysate (**a**) and hemicellulose fraction (**b**) from *F. sylvatica*. Batch fermentation was carried out in a small-scale bioreactor at 35 °C and 300 rpm for 168 h

Since promising results gained with the shake flask experiments with hemicellulose and cellulose from beechwood, batch fermentations in a small-scale bioreactor (vessel volume: 2.0 L) have been performed. The hemicellulose cultivation approaches revealed that the highest yield could be achieved using 99.5 g/L of hemicellulose fraction. Because of this, we used this approach for fermentation. The malic acid and carbon source concentration of beechwood cellulose fraction as well as the malic acid concentration of beechwood hemicellulose fraction during fermentation are shown in Fig. 4.

During fermentation with beechwood cellulose/fiber hydrolysate, malic acid concentration increased to 37.9 ± 2.6 g/L. The carbon source concentration (glucose and xylose) simultaneously decreased from 55 to 16 g/L. This results in a yield of 0.97 g/g (65%). The maximal production rate of 0.78 g/(L * h) was determined in the beginning of the fermentation (0–0.66 h). Maximal volumetric consumption rate (0.26 g/(L * h)) lasted from 160.78 h until the end of cultivation. The production rate of the whole fermentation process was 0.23 g/(L * h).

During fermentation with beechwood hemicellulose fraction, the extended lag phase of about 48 h observed at shake flask cultivation occurred again in bioreactor fermentation. The malic acid concentration increased to a final concentration of 5.8 ± 1.5 g/L resulting in an overall production rate of 0.03 g/(L * h). The maximal volumetric production rate of 0.12 g/(L * h) was from 97.78 to 106.31 h of fermentation time.

### Fermentation of organosolv fractionated lignocellulose with *R. delemar*

**Fig. 5:**
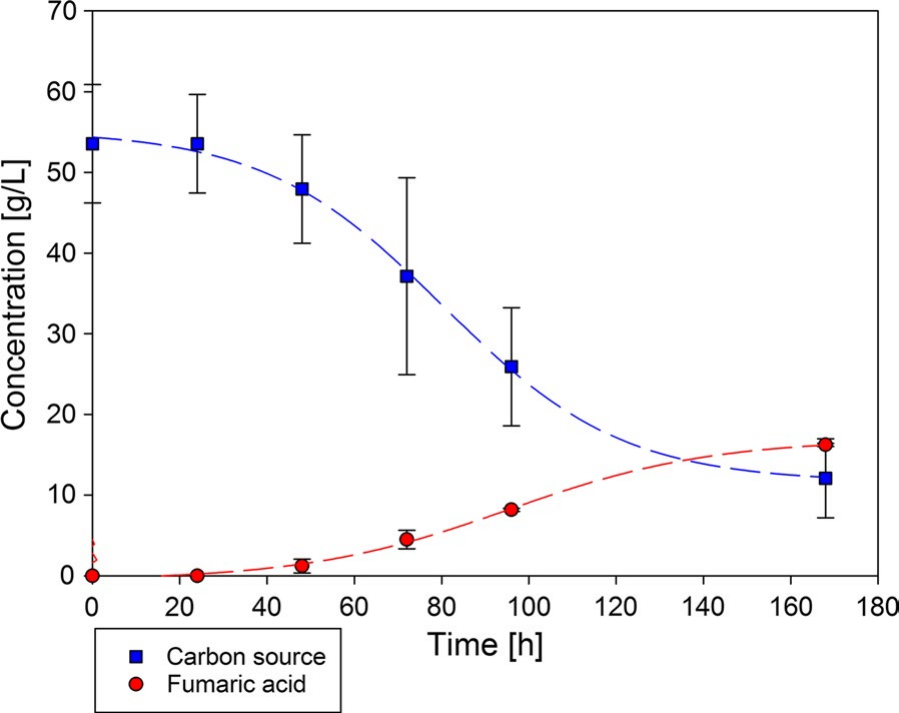
Carbon source and fumaric acid concentration by fermentation of *R. delemar* DSM 905 with cellulose hydrolysate from beechwood. Batch fermentation was done in a small-scale bioreactor at 35 °C and 300 rpm for 168 h

The results of fermentation with beechwood hydrolysates with *A. oryzae* showed the general suitability of this kind of carbon source for fermentation. To demonstrate the suitability of the beechwood carbohydrates as substrate for other fungi, a small-scale batch fermentation was subsequently repeated with the industrial relevant fumaric acid producer *Rhizopus delemar* DSM 905 with the same amounts of organosolv fraction and the same fermentation conditions. Toxicity tests of a selection of possible impurities were done in earlier studies of our group [13] with these fungi. Cultivation with beechwood hemicellulose fraction did not result in product formation. Using beechwood fiber hydrolysate, 16.2 ± 0.2 g/L fumaric acid could be produced. During fermentation, the carbon sources (glucose and xylose) decreased from 53.6 to 12.1 g/L resulting in a yield of 0.39 g/g (30%) and an overall production rate of 0.1 g/(L * h). The results are shown in Fig. 5.

The maximal production rate of 0.19 g/(L * h) was reached after the first half of fermentation (89.91–101.06 h). Maximal volumetric consumption rate (0.53 g/(L * h)) was observed from 76.13 h until 84.66 h.

## Discussion

The number of possible carbon sources for malic acid production with *A. oryzae* is high. The observed flexibility makes this filamentous fungus still interesting for industrial application, although higher yields and titers could be achieved with other fungi like *Ustilago trichophora* [26]. For industrial application the main focus is the price of the carbon source, the productivity, and the yield of the process. Fructose and maltose, both commonly used in food industry, were proved to be very good sources for malic acid fermentation. Both sugars resulted in a good product yield; fructose even results in the highest yield of all tested sugars. The glucose dimer maltose led to the second highest malic acid concentration of 34.2 g/L. Besides most other microorganisms, fungi are only able to consume simple molecules like monosaccharides and amino acids but they are able to secrete enzymes to cleave more complex structures externally. The rate-limiting step of the metabolization of disaccharides of fungi is the extracellular cleavage of the α/β − 1 → 2-glycosidic bond. Because the disaccharide concentration does not reflect the concentration of metabolizable glucose concentration in the media, calculated yield seems too low and consumption rate too high. As all other hexoses can easily be converted to glucose-6-phosphate to enter the glycolysis, the metabolic pathway for galactose is more complex. Although *A. oryzae* expresses the enzyme galactose-1-phosphate uridylyltransferase, an important enzyme for galactose metabolism, and we observed a decrease of galactose during cultivation, the conversion to malic acid seems not possible. Regarding the food vs. fuel debate, the focus of this study was to identify suitable non-edible carbon sources. To achieve a high productivity of the process, a high volumetric production rate is striven. This high production rate, reached in an early stage of fermentation over a long term, is the ideal case. Both parameters vary highly between the sugars investigated. As observed, the time of maximal consumption rate (mostly at the beginning of the fermentation) and the time of maximal production rate (mostly in the middle or end of fermentation) are not correlating with each other. Glucose as the control forms an exception. This is due to the adaptation of the fungus to the respective carbon source, which is not happening by cultivation on glucose because of the pre-culture cultivation on glucose. The longest phase of the highest production rate was observed in cultivations with levoglucosan. The ability of A. *oryzae* to metabolize and produce malic acid from this anhydrosugar was not described before, but it is shown for citric acid production with *A. niger* [27] and itaconic acid production with *A. terreus* [28]. We demonstrated that *A. oryzae* can convert levoglucosan to malic acid with a yield of 0.34 g/g to a final titer of 17.2 g/L. Both are about half of the values obtained for glucose. Like glucose, levoglucosan will be converted to glucose-6-phosphate as the first step of the metabolic pathway. Because of the higher *K*_m_ value of the levoglucosan kinase compared to the hexokinase, this difference can be explained by activity differences [29]. Energetic differences in levoglucosan metabolization (like ATP consuming transport systems) are speculative and not known until now. Nevertheless, pyrolytic sugar is a possible future carbon source.

The other tested sugars are all contained in organosolv-pretreated and fractionated lignocellulose. They showed a very diverse suitability as carbon source. The suitability of xylose as carbon source for malic acid production has already been shown by Ochsenreither et al. [11] and could be verified in this study (0.44 g/g yield). The main components in lignocellulose are by far glucose and xylose. Glucose is the established carbon source for this fermentation process, but in its dimeric form in organosolv-derived pretreated cellulose, cellobiose is a challenge for the organism. The results in Table 2 showed a general suitability, but the resulting yield and titer are in a very low range (0.14 g/g and 8.8 g/L). As well as for maltose, the calculated yield and consumption rate is related to the disaccharide cleavage and does not reflect the real values adequately. However, it shows the better adaption of *A. oryzae* to a starch-containing substrate compared to a lignocellulosic substrate. In preparation for fermentation with enzymatic treated organosolv fractions, mixtures of glucose and xylose were tested. Product titer for both tested mixtures and the yields differ greatly (75% glucose: 29.4 ± 1.9 g/L, 0.38 g/g and 25% glucose: 31.9 ± 0.3 g/L, 0.59 g/g). As observed, *A. oryzae* prefers to metabolize glucose first, until the concentration decreases under a certain level (about 20 g/L). Below that threshold value xylose also gets metabolized. This double usage of glucose and xylose is described for other organisms like *Clostridium* sp. (Strain BOH3) [30] for butanol production and for the yeasts *T. cutaneum* [31] for lipid production. In the 75% glucose and 25% xylose approach, this adaption process has to be done in the middle of the fermentation. Compared to this, in the 25% glucose and 75% xylose approach it happened already during the adaption process of the fungus to the conditions in the main-culture medium. This double usage of sugars could be one of the reasons why yield for the approach with 25% glucose and 75% xylose is much higher than the opposite around. Interestingly, this does not affect the malic acid production rate.

The logical next step was the cultivation on lignocellulosic carbon sources. In the past, fermentation approaches with pretreated lignocellulosic biomass were mostly done for ethanol production for example with bacteria like *E. coli* KO11/SL40 or *Zymomonas* CP4 (pZB5) summarized by Rodney et al. [32] and yeasts like *S. cerevisiae* [33–35], *S. passalidarum* [36], and *P. stipitis* [37]. There is also an approach for the direct conversion of wheat straw without pretreatment with the cellulolytic strain A. *oryzae* A-4 *A*. In this experiment, a lipid yield of 62.87 mg/g dry substrate could be achieved [38]. Approaches for the production of value-added substances are quite low. One of the challenges using organosolv fractions as carbon source is impurities formed during the process, as summarized by Jönsson et al. [18] on the one hand. On the other hand, not focused in this study, the purification of the products is much more complicated using this complex carbon source.

For the majority of the fermentation processes, either an elaborate detoxification process is required or the organism has to be adapted through strain development [39]. Because of the high tolerance level against toxic impurities of *A. oryzae* [13], this organism is predestinated for this kind of carbon source.

Comparing the shake flask cultivation of *A. oryzae* with *F. sylvatica* fiber hydrolysate with *M. giganteus* fiber hydrolysate showed very different results. The starting concentration of glucose and xylose in both approaches differ greatly between 100 and 60 g/L. The volumetric production rate for beechwood cellulose fraction reaches its maximum in an early stage of fermentation, and after most of the sugars are metabolized the malic acid concentration subsequently converges to a limit. In comparison, the *M. giganteus* cellulose hydrolysate cultivation shows a linear decrease of sugars, and the highest production rate at the end of cultivation, when sugar is nearly consumed. This indicates possibly non-detectable, but convertible carbon sources in this approach.

The lag phase of malic acid concentration of about 48 h by cultivation with hemicellulose fraction of beechwood can be explained with a necessary fungal adaptation to the media composition. By comparing the yield (0.42 g/g) of the 99.5 g approach to the yield with pure xylose (0.49 g/g), it is found that they are very similar. A detailed look on the composition of this fraction shows that about one-quarter of the carbohydrates are oligoxylose. *A. oryzae* is described as xylanolytic strain [40], and hence the adaption time can be explained with delayed gene regulation expressing enzymes capable of xylan degradation. The dilution approaches (49.8 and 24.9 g) led to a decrease of the yield. On the one hand, it is known that high amounts of carbon source support the malic acid formation [11] resulting in a lower yield for lower carbon source concentrations. On the other hand, the possible impurities in this fraction seem to be not above an inhibitory limit. Even the main impurity, furfural which is present in the fermentation medium with 0.45 g/L (0.045%), the inhibiting concentration of 0.7% was not reached [13].

The scale-up of the beechwood fiber fraction led to a similar malic acid concentration curve. Maximal volumetric production rate is with 0.78 g/(L * h) higher than in the shake flask experiments, but this value is only achieved in the very beginning of the fermentation process, and decreased after 40 min of fermentation. Carbon source consumption is significantly slower and a higher concentration of sugars is left at the end of the process. This resulted in a higher yield in the bioreactor approach and could be triggered by providing the optimal conditions, in case of oxygen supply and homogenous mixing of the cultivation broth. In all cultivations with beechwood cellulose fraction, there is an increase of carbon source from the beginning of the cultivation to first sample after 24 h, with a subsequent decrease. Most possible reason is the cleavage of oligosaccharides, because only mono- and disaccharides were measured. The scale-up of the *F. sylvatica* hemicellulose fraction differs even more from the shake flask cultivation. Less than half of the amount of malic acid was produced in the bioreactor process. Conceivable is a reaction of impurities to more toxic compounds because of the better oxygen input. Compared to shake flask cultivation, greater amounts of foam were produced during fermentation and must be treated with antifoam. Further a negative impact of the bioreactor conditions for the oligoxylose digestion is possible, but not yet described and because of the xylose detection problems in this fraction being not provable.

For fermentation of lignocellulose fractions with *R. delemar*, no pure sugar conversion experiments are necessary. As a well-known fumaric acid producer, a lot of studies were done with several alternative carbon sources, including different waste products and hydrolysates from lignocellulose [41–46]. *R. delemar* is able to convert xylose, and is also described as xylanolytic [47]. As this fungus is more sensitive towards inhibiting compounds than *A. oryzae* [13], lacking fumaric acid production with *F. sylvatica* hemicellulose fraction is a consequence of the possibly higher amounts of impurities. The beechwood cellulose hydrolysate fits well as carbon source for fumaric acid production. The achieved product concentration of *R. delemar* DSM 905 was even slightly higher with the hydrolysate than with refined glucose (13.1 ± 1.6 g/L, 0.26 g/g, 20%). As shown in further studies of our group, small amounts of phenols can support the organic acid production, which could be the reason for this [13].

## Conclusions

We showed in this study that the range of convertible sugars for *A. oryzae* is even higher than known before. Besides glucose, fructose and maltose could be pointed out as a promising carbon source derived from first-generation renewable resources. Regarding to the “food or fuel” debate, a biotechnological malic acid production based on alternative carbon sources not competing with food or feed production would be desirable. Lignocellulose, as an abundant renewable resource of the second generation, is easily available but its microbial accessibility is a challenge. The anhydrosugar levoglucosan, derived from cellulose during flash pyrolysis, could be figured out as a suitable carbon source. We approved the suitability of fiber/cellulose hydrolysate of the plants *F. sylvatica* (beechwood) and *M. giganteus* obtained from the organosolv process as carbon source for *A. oryzae* in shake flasks as well as in a small-scale bioreactor. Additionally, the more challenging hemicellulose fraction of *F. sylvatica* was also positively evaluated for malic acid production with *A. oryzae*. Both fractions of beechwood were also tested as carbon source for the fumaric acid producer *R. delemar*. Hemicellulose fraction of *F. sylvatica* was only suitable for *A. oryzae*.
